# Supplementary material for: Genetic Characteristics and Microbiological Profile of Hypermucoviscous Multidrug-Resistant Klebsiella variicola Coproducing IMP-4 and NDM-1 Carbapenemases
Source: Microbiol Spectr. 2022 Jan 12;10(1):e01581-21. doi: 10.1128/spectrum.01581-21 (PMC8823660; doi:10.1128/spectrum.01581-21)
Supplement: SUPPLEMENTAL FILE 1 — Supplemental material. Download SPECTRUM01581-21_Supp_1_seq2.pdf, PDF file, 0.6 MB [file spectrum01581-21_supp_1_seq2.pdf]

Table S1. MICs for *K. variicola* stain SHET-01

Antibiotic classes	Antibiotic	MIC(μg/ml)	Interpretation[a]
**β-lactams**	cefotaxime	>16	R
	ceftazidime	>64	R
	cefepime	64	R
	cefotetan	>64	R
	ertapenem	16	R
	imipenem	32	R
	meropenem	32	R
	aztreonam	8	I
**β-lactamase inhibitor combinations**	cefoperazone-sulbactam	>128/4	R
	piperacillin–tazobactam	>128/4	R
	ceftazidime-avibactam	>32/4	R
**Sulfonamide**	trimethoprim-sulfamethoxazole	>8/152	R
**Aminoglycosides**	gentamicin	16	R
	amikacin	≤8	S
**Quinolone**	ciprofloxacin	≤0.25	S
**Fosfomycin**	fosfomycin	≤64	S
**Polypeptides**	colistin	0.5	S
**Tetracyclines**	tigecycline	≤0.25	S

Note: [a], R, resistance; I, intermediate resistance; S, susceptible.

Table S2. Genomic features of *K. variicola* stain SHET-01

	Chromosome	pNDM-IMP-1	pNDM-IMP-2
**size (bp)**	5508387	347317	144371
**G+C (%)**	57.36	48.77	50.24
**No. of predicted ORFs**	5159	405	140
**Resistance genes**			
Fluoroquinolone	*oqxAB*	*aac(6')-Ib-cr*	none
Aminoglycoside		*Aph (3'')-Ib, aph (6)-Id, aph (3'')-Ib, aac(6')-Ib-cr, aac (6')-Ib3, aac (3)-IId,aadA16*	none
Rifamycin		*ARR-3*	none
Folate pathway antagonist		*dfrA27*, *sul1*	none
Aminocyclitol		*aadA16*	none
Phenicol		*catB3*	none
Beta-lactam	bla_{LEN17}	bla_{TEM-1B}, bla_{OXA-1}, bla_{SFO-1}	none
Carbapenem		bla_{IMP-4}, bla_{NDM-1}	none
Macrolide		*mph(A)*, *msr(E)*, *mph(E)*	none
Streptogramin b		*msr(E)*	none
Quaternary ammonium compounds		*qacE*	none
**Virulence genes**	enterobactin (*entABCDEFS*, *fes*, *fepABCDG*), aerobactin (*iutA*), salmochelin (*iroN*), KFU (*kfuABC*), ECP (*ecpABCDE*), fimbria type 1(*fimABCDEFGHI*), fimbria type 3 (*mrkABCDFIJ*), urea (*ureABCDEFG*), bioflim (*treC*, *bdcA*, *bssS*, *tabA*), hemolysin III (*yqfA*), LOS (*lpxABCD*, *rffG*)	none	none

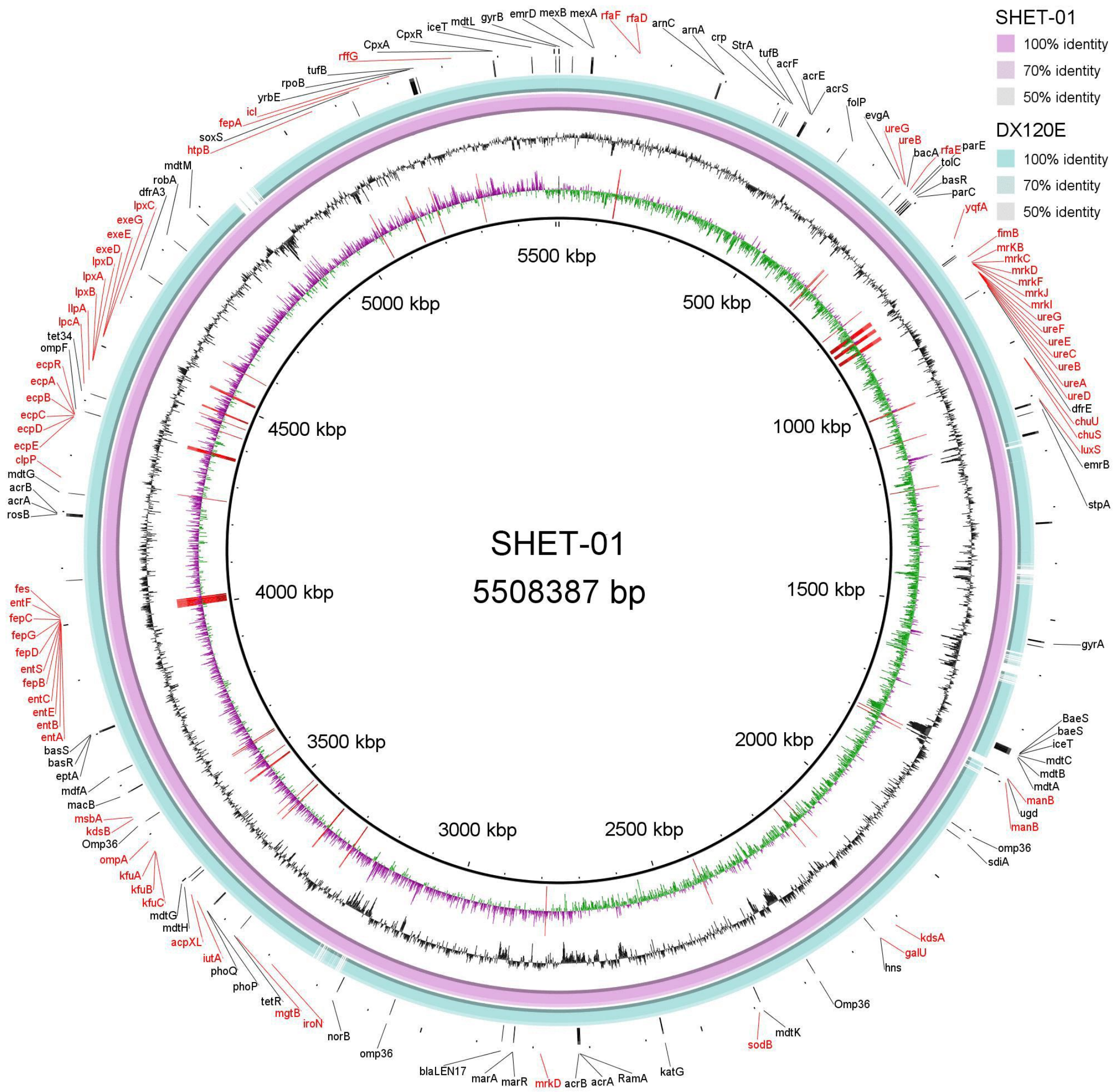

Figure S1. The circle map of the chromosome of strains SHET-01 (CP050680) and DX120E(CP009274.2). The circle map was generated by BRIG tools. Virulence genes are shown in red, and the other resistance-related or functional genes are shown in black.
